# Supplementary material for: Reactive Oxygen Species Mediate Transcriptional Responses to Dopamine and Cocaine in Human Cerebral Organoids
Source: Int J Mol Sci. 2023 Nov 18;24(22):16474. doi: 10.3390/ijms242216474 (PMC10671319; doi:10.3390/ijms242216474)
Supplement: Supplementary file 1 [file ijms-24-16474-s001.zip › Supplemental Material 20231113.pdf]

Supplemental Material

Thomas Rudibaugh, *et al.*

Reactive Oxygen Species Mediate Transcriptional Responses to Dopamine and Cocaine in Human Cerebral Organoids

This file includes:
Figures S1-S4
Tables S1-S3

Not Included:
Supplemental Excel Files 1-7

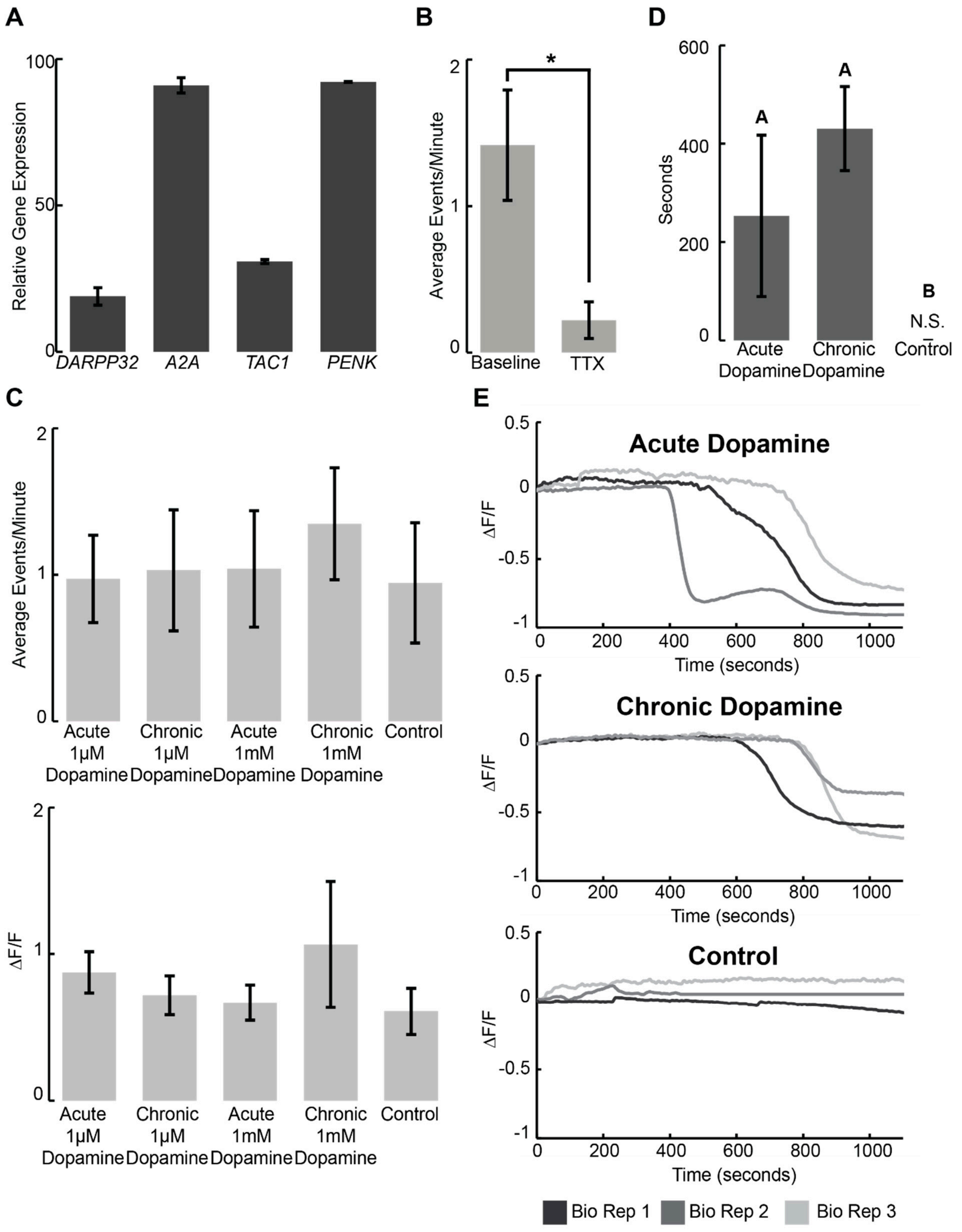
A
Relative Gene Expression
100
50
0
DARPP32
A2A
TAC1
PENK
B
Average Events/Minute
2
1
0
*
Baseline
TTX
D
600
400
200
0
Seconds
A
A
B
N.S.
Acute Dopamine
Chronic Dopamine
Control
C
Average Events/Minute
2
1
0
Acute 1μM Dopamine
Chronic 1μM Dopamine
Acute 1mM Dopamine
Chronic 1mM Dopamine
Control
ΔF/F
2
1
0
Acute 1μM Dopamine
Chronic 1μM Dopamine
Acute 1mM Dopamine
Chronic 1mM Dopamine
Control
E
Acute Dopamine
ΔF/F
0.5
0
-0.5
-1
0
200
400
600
800
1000
Time (seconds)
Chronic Dopamine
ΔF/F
Time (seconds)
Control
ΔF/F
Time (seconds)
Bio Rep 1
Bio Rep 2
Bio Rep 3

Figure S1, Related to Figure 1; ***Ventral forebrain organoids exhibit Ca^{2+} and intracellular cAMP responses to dopamine***

A) RT-qPCR of D90 organoids for MSN markers DARPP32, A2A, TAC1, and PENK. Gene expression was compared to the reference gene GAPDH, and ΔΔCq values were found by comparing against undifferentiated stem cell controls. **B)** Ca^{2+} imaging of D90 organoids shows transients knocked down by TTX. Average transients/minute were measured for active cells (defined as any cell exhibiting at least one Ca^{2+} transient during the measurement period). **C)** Frequency and amplitude of Ca^{2+} transients following acute or chronic 1μM or 1mM dopamine exposure. Measurements were averaged across all active cells. **D)** Response time of intracellular cAMP increase following acute or chronic 1μM dopamine exposure. **E)** Representative response curves of intracellular cAMP increase related to Figure 1F and Figure S1D. **A)** Error bars represent 95% confidence for n=5 replicates. **B-D)** Error bars represent 95% confidence for n=3 replicates. **B-C)** Error bars refer to the mean frequency and amplitude across all cells within an individual biological replicate that display at least 1 calcium transient during the imaging period. **B)** * $p<0.05$, paired t-test. **C)** Statistics: $p<0.05$, one-way ANOVA with Tukey Kramer post hoc analysis. Conditions sharing the same superscript letters are not statistically significant from each other. Therefore if two bars do not share the same lettering, they are statistically significant from each other.

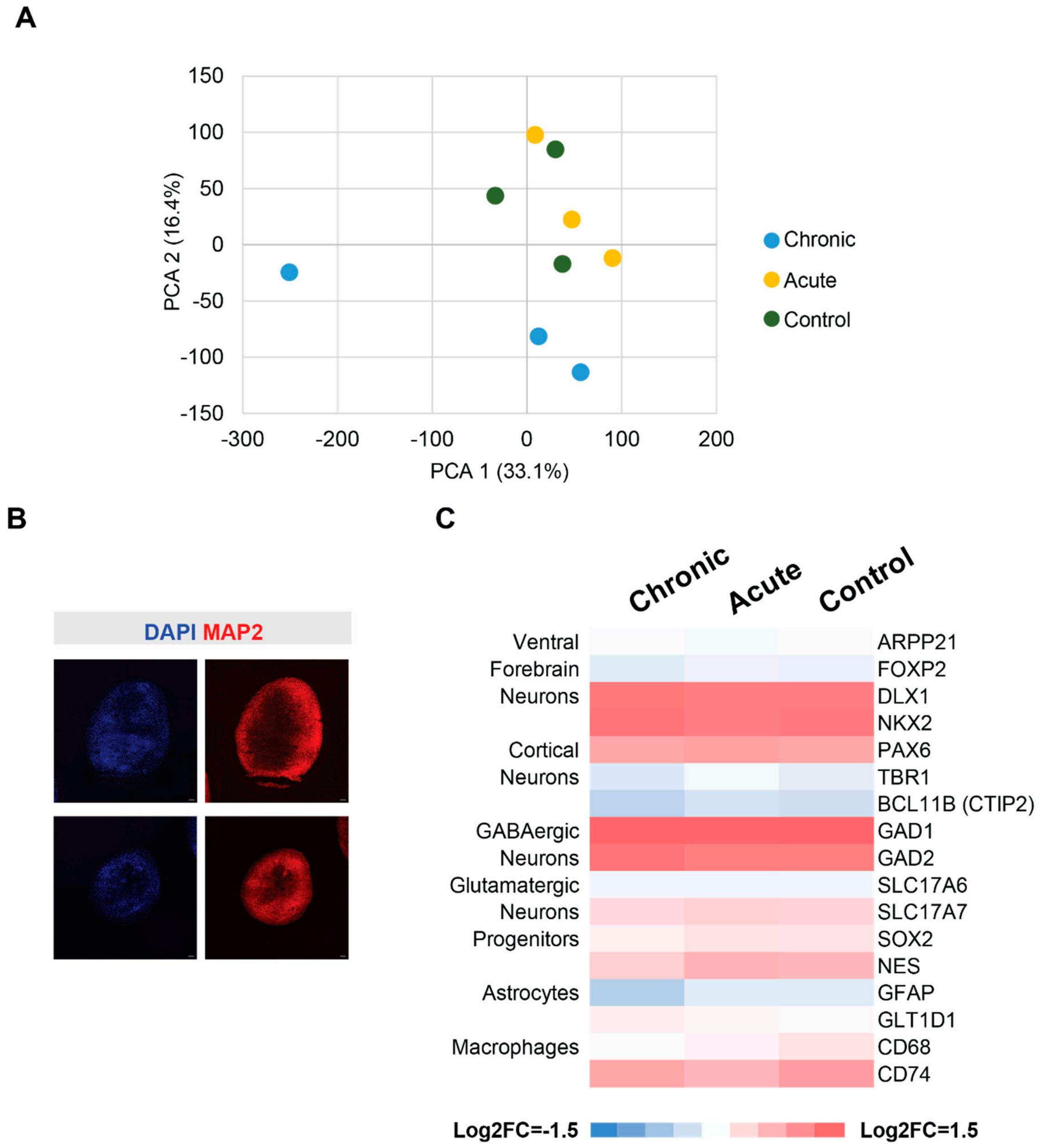
A
150
100
50
0
-50
-100
-150
-300
-200
-100
0
100
200
PCA 2 (16.4%)
PCA 1 (33.1%)
Chronic
Acute
Control
B
DAPI MAP2
C
Chronic
Acute
Control
Ventral Forebrain Neurons
Cortical Neurons
GABAergic Neurons
Glutamatergic Neurons
Progenitors
Astrocytes
Macrophages
ARPP21
FOXP2
DLX1
NKX2
PAX6
TBR1
BCL11B (CTIP2)
GAD1
GAD2
SLC17A6
SLC17A7
SOX2
NES
GFAP
GLT1D1
CD68
CD74
Log2FC=-1.5
Log2FC=1.5

Figure S2, Related to Figure 2- ***Transcriptional analysis reveals immune-related response to acute and chronic dopamine***

A) Principal component analysis of the three biological replicates for the three different conditions: chronic dopamine, acute dopamine, and vehicle control. **B)** Immunostaining images of D90 organoids demonstrating neural marker *MAP2* expression around organoid periphery, but not in organoid core. **C)** Heat map of select genes relative to 12 pcw striatal transcriptome.

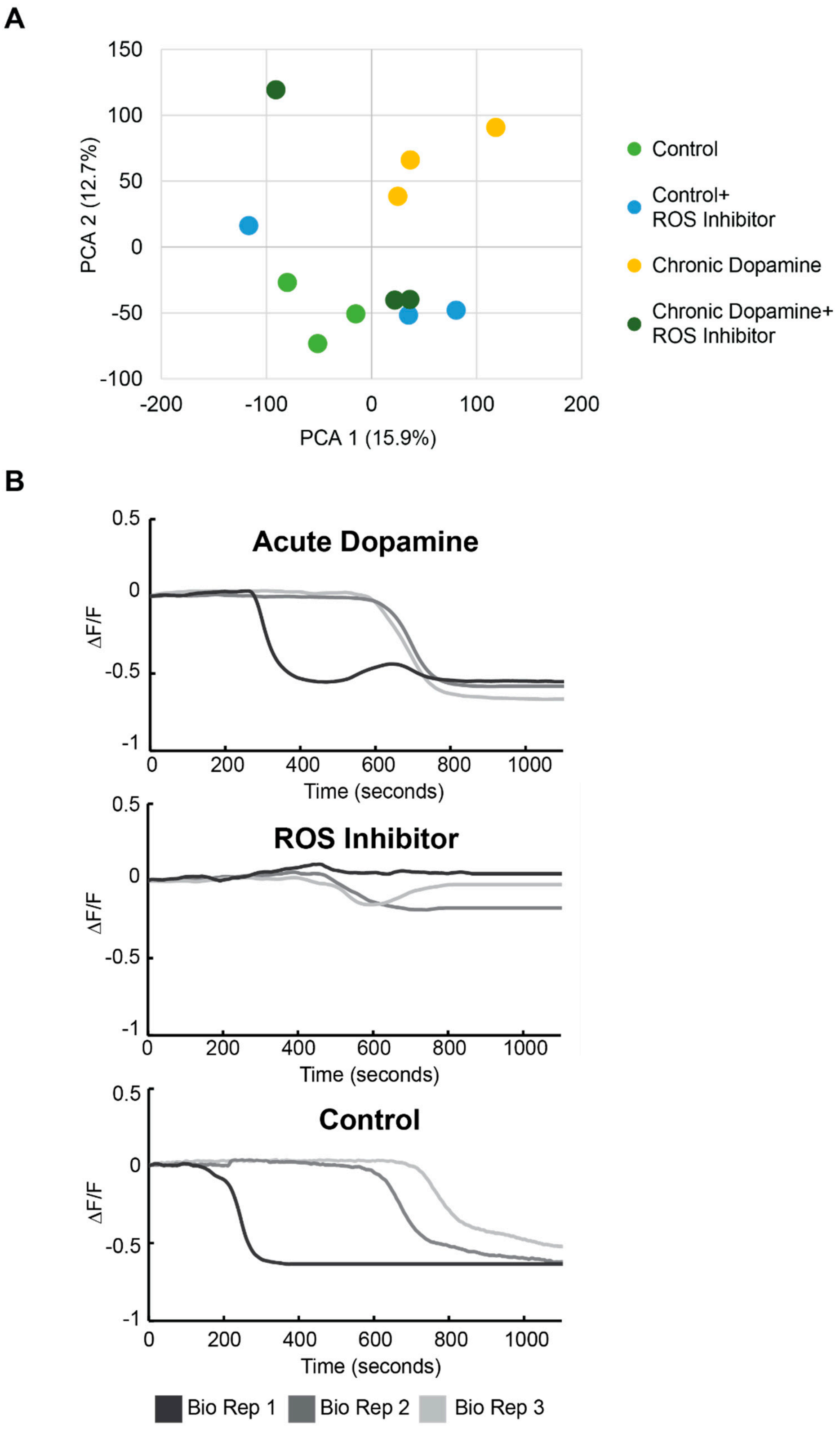
A
150
100
50
0
-50
-100
PCA 2 (12.7%)
-200
-100
0
100
200
PCA 1 (15.9%)
Control
Control+ ROS Inhibitor
Chronic Dopamine
Chronic Dopamine+ ROS Inhibitor
B
Acute Dopamine
ΔF/F
Time (seconds)
ROS Inhibitor
ΔF/F
Time (seconds)
Control
ΔF/F
Time (seconds)
Bio Rep 1
Bio Rep 2
Bio Rep 3

Figure S3; Related to Figure 3- ***Organoid response to dopamine driven by reactive oxygen species***
A) Principal component analysis showing the three replicates for the four conditions: control, control dosed with an ROS inhibitor, chronic dopamine dosed, and chronic dopamine dosed with an ROS inhibitor. **B)** Representative response curves of intracellular cAMP increase related to Figure 3B.

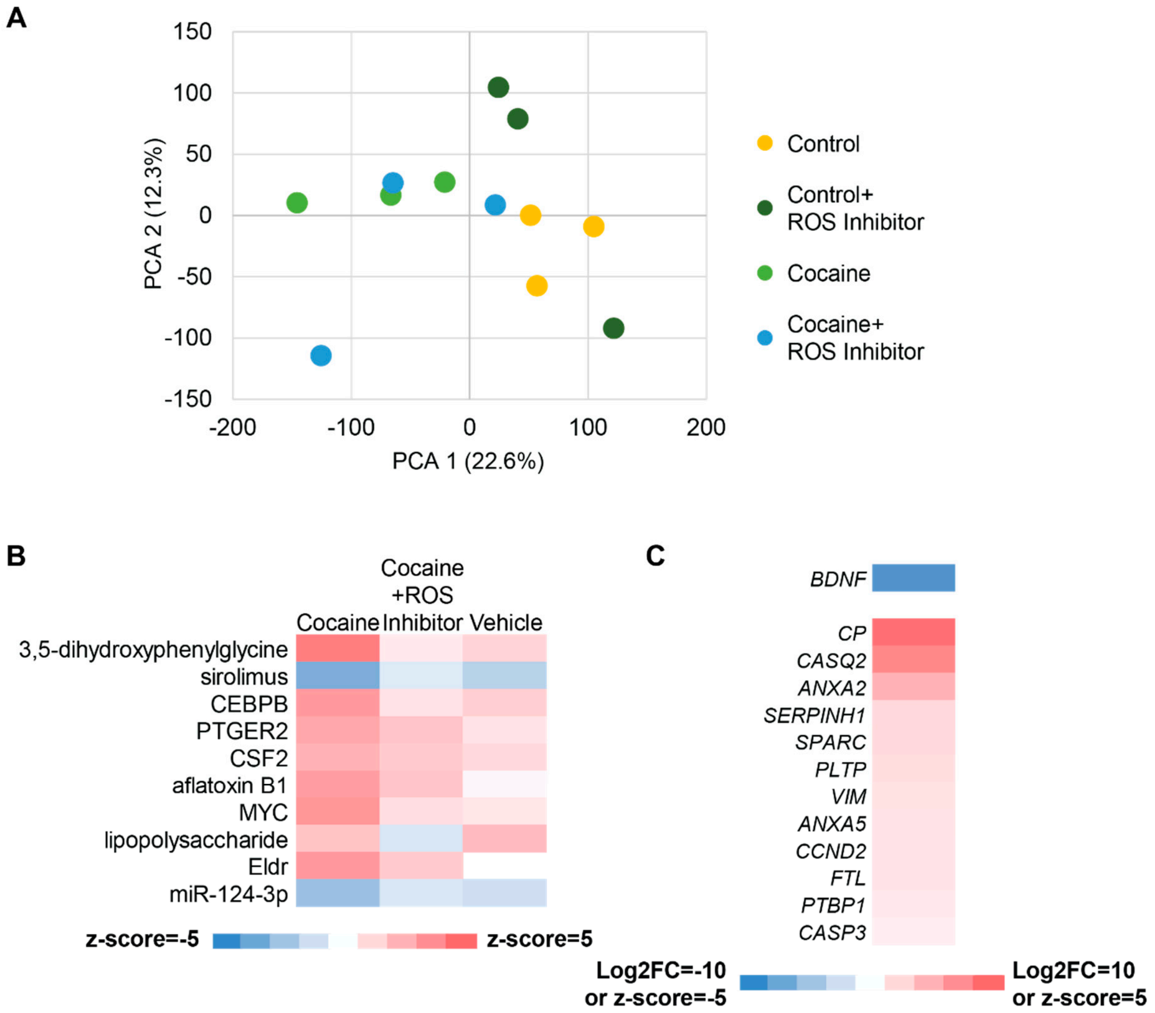

Figure S4; Related to Figure 4- ***ROS also mediates the transcriptomic response to cocaine***
A) Principal component analysis of the three biological replicates for the four dosing conditions: vehicle controls, vehicle controls with an ROS inhibitor, chronic cocaine, and chronic cocaine with an ROS inhibitor. **B)** Heat map of the top regulated upstream regulators by z-score identified using GSEA following dopamine or cocaine exposure with or without an ROS inhibitor. **C)** Heat map of BDNF predicted z-score and Log2FC of genes associated with BDNF downregulation based on Ingenuity Pathway Analysis (IPA) database.

Table S1
Related to Figure 1, Primary Antibodies

Antigen	Species	Company	Catalog	RRID	Dilution
DARPP32	Rabbit	Abcam	Ab40801	AB_731843	1:50
MAP2	Mouse	Sigma-Aldrich	M1406	AB_477171	1:250
GABA	Rabbit	Sigma-Aldrich	A2052	AB_477652	1:200
CTIP2	Rat	Abcam	Ab18465	AB_2064130	1:250
GSX2	Rabbit	Millipore	Abn162	AB_11203296	1:500
SOX2	Goat	R&D Systems	AF2018	AB_355110	1:100

Table S2
Related to Figure 1, Secondary Antibodies

Species	Flourophore	Company	Catalog	RRID	Dilution
Rabbit	488	Life Technologies	A21206	AB_2535792	1:250
Mouse	546	Thermofisher	A10036	AB_2534012	1:250
Rat	647	Jackson Immunoresearch	712605150	AB_2340693	1:125
Goat	647	Jackson Immunoresearch	705605003	AB_2340436	1:125

Table S3
Relative to Figure S1, qPCR Primers

Gene Symbol	Gene ID	Forward Primer	Reverse Primer
DARPP32	84152	TTGGAAAATCCAGAAAACCG	CTGGTAGAAGCCGGTGAGAG
A2A	135	AGGCAGCAAGAACCTTTCAA	CTAAGGAGCTCCACGTCTGG
PENK	5179	GCTGTCCAAACCAGAGCTTC	TCTGGCTCCATGGGATAAAG
TAC1	6863	TGGGGTTGAAAATTCAAAAAG	GGAGTTTCCTTCCTTTTCCG
GAPDH	2597	ATGACATCAAGAAGGTGGTG	CATACCAGGAAATGAGCTTG
